# Responses to Low- and High-Intensity Exercise in Adolescents with Type 1 Diabetes in Relation to Their Level of VO_2_ Max

**DOI:** 10.3390/ijerph18020692

**Published:** 2021-01-15

**Authors:** Artur Myśliwiec, Maria Skalska, Arkadiusz Michalak, Jędrzej Chrzanowski, Małgorzata Szmigiero-Kawko, Agnieszka Lejk, Joanna Jastrzębska, Łukasz Radzimiński, Guillermo F. López-Sánchez, Andrzej Gawrecki, Zbigniew Jastrzębski

**Affiliations:** 1 Department of Physiology, Gdansk University of Physical Education and Sport, 80-336 Gdansk, Poland; admysliwiec@wp.pl (A.M.); lukaszradziminski@wp.pl (Ł.R.); zb.jastrzebski@op.pl (Z.J.); 2 Department of Pediatrics, Diabetology and Endocrinology, Gdansk Medical University, 80-210 Gdansk, Poland; mkawko@gumed.edu.pl (M.S.-K.); agnieszka.lejk@op.pl (A.L.); joanna.jastrzebska@hotmail.com (J.J.); 3 Department of Pediatrics, Diabetology, Endocrinology and Nephrology, Medical University of Lodz, 91-738 Lodz, Poland; arkadiusz.michalak.lek@gmail.com; 4 Department of Biostatistics and Translational Medicine, Medical University of Lodz, 92-215 Lodz, Poland; jj.chrzanowski1@gmail.com; 5 Faculty of Health, Education, Medicine and Social Care, Anglia Ruskin University-Cambridge Campus, Cambridge CB1 PT1, UK; guillermo.lopez-sanchez@aru.ac.uk; 6 Department of Internal Medicine and Diabetology, Poznan University of Medical Sciences, 61-701 Poznan, Poland; pompainsulinowa@wp.pl

**Keywords:** type 1 diabetes, oxygen consumption, blood glucose, exercise intensity

## Abstract

The purpose of this study was to investigate the influence of maximal oxygen uptake (VO_2_ max) on the glycemic changes during low and high intensity exercises in young type 1 diabetic patients. Twenty boys (age: 14.3 ± 1.6 years; height: 171.0 ± 11.3 cm; weight; 59.5 ± 12.8 kg) were divided into low-fit group (LFG, *n* = 10) and high-fit group (HFG, *n* = 10). According to the experimental design, participants performed three physical efforts (VO_2_ max test, mixed aerobic–anaerobic effort and aerobic effort) on the cycloergometer, during which real-time glycemia was measured. Mixed aerobic–anaerobic exercise demanded significantly smaller carbohydrate supplementation (0.2 ± 0.2 g/kg during exercise) than the aerobic test session (0.4 ± 0.3 g/kg during exercise). Moreover, patients with higher VO_2_ max had lower tendency for glycemic changes during the aerobic effort. The results of the current study suggest that young type 1 diabetic patients should perform different intensity activities using continuous glycemic monitoring system to avoid acute and chronic complications of the disease.

## 1. Introduction

Type 1 diabetes is an autoimmune disease that, under the influence of environmental factors, manifests itself in genetically predisposed individuals [1]. The pathomechanism of the disease involves T effector lymphocytes, which destroy the beta cells of pancreatic islets producing insulin. Their destruction usually leads to absolute insulin deficiency and the appearance of clinical symptoms of the disease. Long-term treatment consists of a life-long insulin therapy, which involves subcutaneous insulin administration. In addition to insulin therapy, adequate exercise and a scrupulously maintained diet are necessary and integral components of effective treatment of type 1 diabetes [2]. Factors such as properly planned physical activity and the ability to react to changes in glycemia during the training session provide a substantial chance for safe course of glycemia and the absence of complications after the training. Physical activity performed by patients with diabetes does not have to differ from that of a healthy individual.

The challenge for the patient and the doctor is to adjust these treatment components in a manner that they complement each other, consequently maintaining normoglycemia around-the-clock and minimizing the patient’s glucose fluctuations [3,4,5,6].

In recent years, there has been an increase in the incidence of type 1 diabetes in children and adolescents [7]. Diabetic societies recommend physical recreation and even sports as a part of treatment that could have positive influence on acute and chronic vascular complications prevention [8,9,10]. There are many examples of top-level athletes in whom type 1 diabetes did not prevent them from achieving spectacular sports successes (Sir Steve Redgrave, a five-time Olympic gold medalist in rowing; Kris Freeman, a four-time participant in cross-country skiing at the Winter Olympics; and American swimmer Gary Hall, a gold medalist at the Athens Olympic Games) [10,11]. As a result, there is an increasing demand for new therapeutic solutions that will enable patients to engage in physical activity, including sports, safely.

Understanding the influence of exercise on glycemic profiles during and after exercise, it is crucial to optimize insulin doses. Moreover, therapeutic decisions should be made in accordance with metabolic changes in the body under the influence of different intensity [12]. Chimen et al. [13] emphasized the positive effects of physical activity on lipid profile, endothelial function and insulin sensitivity in patients. Many papers assess the effect of exercise on supporting the treatment of type 2 diabetes mellitus [14,15,16,17,18]. However, few paper discuss this issue for type 1 diabetes in children [4,10,19].

The influence of physical exercise on blood glucose level and its fluctuation during and after the exercise is a complex issue. Yardley and Sigal [20], Ramalho et al. [21] and Reddy et al. [22] showed that the body’s response to physical effort in relation to changes in glycemic fluctuation depends on the type of physical activity undertaken. High- intensity effort (aerobic or anaerobic) lasts several minutes, is carried out with the use of repeated or interval training and is performed with high power output [23].

Maximal oxygen uptake (VO_2_ max) is one of the most popular and widely used indexes describing the level of physical fitness. Higher values of VO_2_ max allow for faster and more effective recovery after the physical exercise. Thus, an appropriate VO_2_ max value together with neuro-muscular adaptations and correctly planned training process enable better adaptation to large physical loads. On the basis of the VO_2_ max, it is possible to determine the intensity of exercise (%VO_2_ max) [24].

According to Scott et al. [2], physical exercise provides metabolic challenges for type 1 diabetic patients. Maintaining the proper level of blood glucose concentration before, during and after exercise is dependent on insulin dosing strategy and carbohydrate intake. Factors such as exercise intensity, duration, nutritional status and training status influence the risk of glycemic disturbance. Patients with type 1 diabetes, who do not produce insulin, are deprived of the endogenous insulin regulation responsible for maintaining a constant level of glycemia. Consequently, when muscles have completely used up their glycogen resources, their cells start to draw energy for further exercise from glucose, directly from the bloodstream. Then, with a lack of external carbohydrate supply and a high concentration of exogenous insulin, which prevents the “release” of liver glycogen and gluconeogenesis from fatty acids, there may be a dramatic drop in blood glucose levels. Other metabolic reactions occur in the body during mixed (aerobic–anaerobic intensity) exercise, especially with the predominance of anaerobic metabolism. High-intensity exercise leads to hyperglycemia, which is usually recorded shortly after exercise. This phenomenon is explained by the increased concentration of hormones secreted under the influence of intense, short-term effort: adrenaline, cortisol, glucagon and growth hormone [25].

The aim of our experiment was to demonstrate that the level of VO_2_ max in young patients with type 1 diabetes can have a significant effect on the regulation of glycemic changes during low and high intensity exercise. It was hypothesized that patients with higher levels of VO_2_ max would respond to applied efforts significantly better with lower glycemic fluctuation during exercise and rest. Moreover, they would experience fewer hypoglycemic incidents as delayed reactions to physical effort during the recovery phase.

## 2. Materials and Methods

### 2.1. Characteristics of the Study Group

The study was composed of 20 boys with T1D diagnosed according to the criteria of ISPAD guidelines [26], who remained under the care of the Clinic of Pediatrics, Diabetology and Endocrinology at the University Clinical Center in Gdańsk, Poland, a city located at latitude 54°22′ north (mean age: 14.3 ± 1.6 years, height: 171.0 ± 11.3 cm; weight: 59.5 ± 12.8 kg; mean diabetes duration: 6.7 ± 4.1 years; mean HbA1c: 7.3 ± 0.8%). Researchers obtained the approval of the Bioethical Commission of the Medical University of Gdansk (NKBBN/397/2018). The protocol of the study was explained to every participant before enrolment. Patients and their parents signed the written informed consent form. Inclusion criteria of the study were: assessment of the puberty at Stage III–V in the Tanner scale; not practicing high performance sports; lack of diabetic ketoacidosis or severe hypoglycemia incidents during last five years; and signed written consent form. Exclusion criteria were: obesity; concomitant chronic diseases (e.g., hypothyroidism, liver or renal disorders and celiac disease) that may have an impact on the occurrence of hypoglycemia; or the lack of written informed consent.

Patients were using insulin pump therapy integrated with continuous glucose monitoring (CGM). Medtronic sensor and insulin pumps, Paradigm Veo and MiniMed 640G, were used in the study. CGM measured real-time glycemia and was not blinded, therefore the researchers and patients had continuous access to glucose concentrations. The detailed clinical and laboratory characteristics of the group are presented in Table 1. All patients were in the pubertal period (no patients in Tanner Stage I) with majority being in Stages III (60%) and IV (25%). No participants were obese but two (10%) were slightly overweight. According to PAQ (Physical Activity Questionnaire) [27], half of the children reported adequate physical activity for their age (N-10, 20%), seven (35%) were found to be insufficiently active and three (15%) exhibited high physical activity profiles. Mean VO_2_ max capacity measured in all examined patients was 40.2 ± 5.8 mL/kg/min. The subsequent division was based on VO_2_ max median to avoid influence of possible outliers, namely those with VO_2_ max ≤ 41.3 mL/kg/min were defined as low fit group (LFG, lower VO_2_ max) and those with VO_2_ max > 41.3 mL/kg/min were defined as high fit group (HFG, higher VO_2_ max). Interestingly, questionnaire-based assessment was not consistent with VO_2_ max-based labels, with corresponding Cohen’s kappa 0.10. For the following comparisons, we consequently used division into lower VO_2_ max (LFG) and higher VO_2_ max (HFG).

### 2.2. Experimental Protocol

The experimental protocol consisted of three exercise tolerance tests. The objective of the first test was to determine the maximum capacity of every participant. The second test (30 min) was based on intensity of aerobic–anaerobic metabolism (mixed). In the third test (45 min), intensity corresponded to aerobic metabolism (aerobic).

All patients consumed the same meal (natural yoghurt, oat flakes, banana and walnuts) 2 h before each exercise tolerance test. It was composed of 60% carbohydrates, 15% proteins and 25% fat. Forty grams of natural yoghurt, 15 g of oat flakes, 40 g of unripe banana and 4 g of walnuts were included in two carbohydrate units (CU). The amount of CU depended on the total daily caloric requirement of the patient and participants received 0.7–0.8 units of insulin for every CU and every fat/protein unit (FPU).

Each participant received a bolus of rapid-acting insulin analog before the meal, which did not affect glycemia during the physical activity regarding the time of action of the drug. Blood glucose concentration did not exceed 150 mg/dL before enrolment to the aerobic physical exercise and 180 mg/dL before the commencement of the aerobic–anaerobic exercise. Glucose concentrations were controlled with the use of CGM, under medical supervision, during each physical tolerance effort, 1 h after its completion and onwards up to 24 h after the experimental effort.

During the first experimental effort, all of the participants accomplished the progressive load physical tolerance test after preliminary medical procedures. On the basis of obtained results, researchers estimated the individual value of maximum oxygen consumption (VO_2_ max) and anaerobic threshold (AT) rate expressed in power units on a bicycle ergometer. These ratios were essential to determine an individual load as a relative value of AT in subsequent physical tests. Two weeks later, the patients were subjected to 30 min aerobic–anaerobic (mixed) physical activity test, which consisted of alternative bouts of exercise: 2 min of work at AT−40% [Watts] and 4 min at AT + 10% [Watts] (5 repetitions altogether). Two weeks later, the 45 min aerobic physical activity test was used after preliminary medical procedures. The load was expressed in power units (Watts) and the value was determined as 60% of AT (Watts). Cardiopulmonary indices were continuously registered during all physical tolerance tests with the use of an expiratory gas analyzer.

### 2.3. Physiological Analyses

VO_2_ max was measured with the use of expiratory gas analyzer Oxycon Pro (Erich JAEGER GmbH, Hoechberg, Germany, 2012). Experimental runs were performed in a physical effort laboratory in standard conditions (temperature: 21 °C; atmospheric pressure: 1010 hPa; air humidity: 55%) according to previously described procedures [28,29]. Physical tolerance test was preceded by 5 min warm-up in the form of an ergometric work (Eos Sprint, Jeager, Hoechberg, Germany) with the load of 1 W/kg, at the rate of 60 rotations per minute. Subsequently, starting with the sixth minute of the test, the load was increased every minute by 0.25 W/kg. The physical activity was interrupted when the rotation rate decreased by more than 10%, i.e., less than 54/min. The highest relative oxygen consumption, maintained for 15 s, at the end of the exercise was considered as VO_2_ max. The anaerobic threshold was determined by the analysis of expiratory gases exhaled during exercise tolerance test. AT was calculated as a quotient of carbon dioxide exhalation and oxygen consumption (respiratory exchange ratio (RER)). When the calculated value was ≥1, it was assumed that the AT was reached. After experimental runs, patients rested sitting for 5 min and the expiratory gas analyzer was detached.

### 2.4. The Control of Blood Glucose Concentrations

Raw data from CGM were downloaded and trimmed to include only measurements from the exercise days. Only records with >70% of expected measurements were accepted for analysis. Glycemic control parameters (daily blood glucose concentration including during tests) were calculated with Glyculator 2.0. [30]. The insulin dose was assessed as daily insulin dose (DDI) per kg of body weight (IU/kg).

### 2.5. Biochemical Analyses

Laboratory tests were performed in the accredited Central Laboratory of University Clinical Centre in Gdańsk. The biological material for the study was venous blood collected from fasting patients just before the tests. HbA1c was determined by high-performance liquid chromatography (HPLC) using the Bio-Rad VARIANT™ HbA1c Program (Bio-Rad Laboratories, Inc., Hercules, CA, USA), with its values represented as percentages. The level of glycated hemoglobin (HbA1c was determined by high performance liquid chromatography (HPLC) using the Variantfrom BioRad. Total cholesterol (115–190 mg/dL), HDL cholesterol (>40 mg/dL) and triglycerides (<150 mg/dL) were measured on an Alinity analyzer (Abbott, Wiesbaden, Germany) using the enzyme-linked immunoassay method, while LDL cholesterol (<115 mg/dL) was calculated using the Friedewald formula. The levels of thyrotropic hormone (TSH, 0.35–4.94 uU/mL) and free thyroxine (FT4, 9.01–19.05 pmol/L) were determined by a two-stage immunochemical method using microparticles and a chemiluminescent marker from Abbott, Germany. Alanine (<55 U/L) and asparagine aminotransferase (5–34 U/L) were determined by spectrophotometry using an Abbott Alinity analyzer (Wiesbaden, Germany).

### 2.6. Statistical Analyses

Continuous variables are presented as means ± standard deviations. Comparisons between two groups were made using t-test for independent samples for most variables; however, for some, the differences were tested with Mann–Whitney U test (marked by #) due to lack of normal distribution. The differences between multiple subgroups were tested with ANOVA containing repeated measures component. The main tested effects included exercise type (treated as repeated measure for each participant) and VO_2_ max category. Interactions between those effects were also tested, but they are reported only in the case of significant findings. In the case of significant global tests (for main effects or interactions), post-hoc comparisons were made with Bonferroni tests.

## 3. Results

All patients successfully completed all three tests sessions. We observed no severe hypoglycemia episodes or DKAs (diabetic ketoacidosis) before (within 48 h), during or shortly after (within 48 h) the sessions. However, a portion of patients experienced technical issues with their CGM devices (most often sensor detachment during exercise or resting period due to profuse sweating). Therefore, an analysis of CGM-based metrics was limited to those boys who provided >75% complete data for each analyzed period.

Initial insights based on recorded SMBG (self-monitoring of blood glucose) and consumed carbohydrates are presented in Table 2. Briefly, all patients were similarly prepared for the test sessions, as evidenced by lack of significance in pre-exercise amount of consumed carbohydrates. This resulted in starting glucose levels being in target for all test sessions, comparable between tests (maximum: 156.7 ± 38.4 mg/dL; mixed: 157.3 ± 30.6 mg/dL; aerobic: 141.9 ± 25.1 mg/dL, *p* = 0.2663) and less and more fit participants (155.0 ± 31.8 mg/dL vs. 148.9 ± 32.6 mg/dL, *p* = 0.4447). During and after exercise, the patients consumed varied amounts of carbohydrates, significantly different for each type of effort (*p* = 0.0012). Overall, the short test at maximum capacity demanded the smallest supplementation during the exercise (0.1 ± 0.3 g/kg), followed by mixed aerobic–anaerobic exercise (0.2 ± 0.2 g/kg) and aerobic test session (0.4 ± 0.3 g/kg) with significant post-hoc difference between maximum mixed and aerobic exercise tests. Despite differences in carbohydrate consumption, glucose levels at the end of tests (*p* = 0.0011) as well as after 5 min resting period (0.0021) were significantly different for each test type, reaching lowest values after aerobic session. In these analyses, the level of patient VO_2_ max had no statistically significant effect on amount of consumed carbohydrates or end-of-tests sessions blood glucose.

The results are presented as means ± standard deviations. The differences were tested with ANOVA containing repeated measures component (for effort type) and VO_2_ max category effect. *p*-values for each effect are shown on the right-hand side. In the case of significant global test, post-hoc comparisons were made with Bonferroni tests. Significant pairs are denoted by # and $ superscript. Subgroup interactions were also investigated but are omitted for clarity. There were no significant interactions between efforts type and VO_2_ max capacity.

Afterwards, we investigated high-resolution CGM records for those with available data. These were 14–16 participants depending on the comparisons. The dropouts were mostly due to technical issues with CGM devices (most often sensor detachment during exercise or resting period due to profuse sweating) and were not preferential to either studied group.

**Figure 1:**
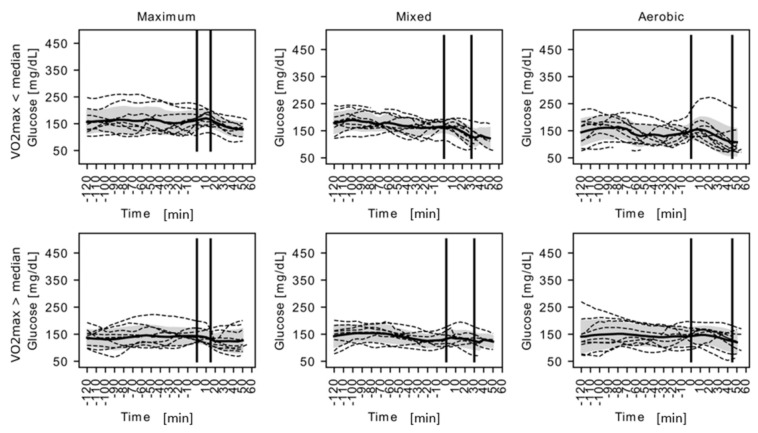
CGM traces for all study participants during each test effort.

CGM analysis revealed that the high VO_2_ max group displayed tendency toward lower mean blood glucose during 2 h preceding exercise sessions (139 ± 33 mg/dL vs. 162 ± 35 mg/dL, *p* = 0.0584, *n* = 8 in each group) and significantly lower blood glucose standard deviation in the same period (15 ± 8 mg/dL vs. 22 ± 10 mg/dL, *p* = 0.0312, *n* = 8 in each group), presenting overall more stable glucose profiles (Figure 1). 

**Figure 2:**
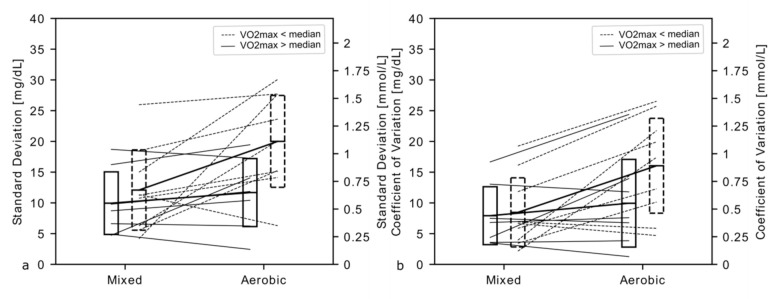
Effect of VO_2_ max on glycemic variability during mixed and aerobic workout: (**a**) glucose standard deviation; and (**b**) coefficient of variation.

Figure 1 shows CGM traces for study participants with available good-quality records (*n* = 16) during each effort. Each dashed line depicts one individual CGM record; bolded lines show mean glucose for each group at each timepoint; and the shaded area covers group glucose standard variation. Vertical lines the denote start and finish of each test effort. The figure is only a graphical representation. Formal statistical comparisons are included in the main text and in Figure 2.

The effect of VO_2_ max on glycemic variability ((a) glucose standard deviation; and (b) coefficient of variation) during mixed and aerobic workout is shown in Figure 2.

Given the short duration of the maximum capacity, it was not possible to quantify glycemic variability (SD or CV) during the exercise. However, the mixed and aerobic sessions differed significantly in this regard (SD of mixed: 11.2 ± 6.2 mg/dL vs. aerobic: 16.4 ± 8.2 mg/dL, *p* = 0.0155; CV of mixed: 8.2 ± 5.4% vs. aerobic: 13.4 ± 8.3%, *p* = 0.0052). HFG displayed tendency toward lesser increase in glucose variability during aerobic session; however, in this limited group, the subgroup interactions did not reach significance (Figure 2).

Maximum capacity effort was excluded from this analysis due to its short duration. Horizontal lines represent means, while boxes denote standard deviation around the mean. The raw values for each patient are linked by slanted lines, denoting individual direction of changes between the tests. Dotted lines pertain to patients with low VO_2_ max while solid ones to the HFG group. It can be seen that patients in LFG present higher glycemic variability during aerobic sessions. However, these differences did not reach statistical significance (*p* for interaction in ANOVA: 0.0984 for SD and 0.0765 for CV), hence no post-hoc tests were performed.

Overall, during or immediately after tests, we noted no events of clinically-relevant hypoglycemia (blood glucose < 54 mg/dL) and few hypoglycemia-alert values (blood glucose < 70 mg/dL) (*n* = 5/60, 8.3%). No alert values were measured during maximum test (0/20, 0%), one was detected during mixed session (1/20, 5%) and four occurred during aerobic exercise (4/20, 20%); however, these differences in frequencies did not reach statistical significance (*p* = 0.05864). Two events occurred in patients from LFG group and three in those from HFG group; however, the difference could not be appraised statistically. Out of those episodes detected by SMBG, 2/5 were missed by CGM monitoring and one more was undetected due to sensor detachment. On the other hand, CGM detected one transient fall in glucose during aerobic exercise that was missed by SMBG control (SMBG: 76 mg/dL; CGM: 68 mg/dL).

## 4. Discussion

The experiment involved 20 adolescent boys with type 1 diabetes. Due to the different VO_2_ max, the whole group was divided into those with lower (LFG) and higher (HFG) levels of physical performance. Both subgroups of patients were homogeneous in terms of their biological development, biometric indicators and physical capacity (VO_2_ max). The main finding of this study is that adolescent type 1 diabetes with higher level of maximal oxygen uptake had a lower tendency for glycemic changes during the aerobic effort. Moreover, the continuous glycemic monitoring system is a useful tool to avoid acute and chronic complications during the physical exercise.

The aim of the experiment was to demonstrate that the level of physical performance determined by the VO_2_ max index can have a significant influence on the glycemic fluctuation in the examined patients during their mixed and aerobic exercise. Moreover, patients with higher VO_2_ max will have fewer hypoglycemic incidents after exercise. In the available literature, there is a lack of publications determining the correlation between physical performance in children and young people with type 1 diabetes and their tolerance to physical effort of varying intensity with regard to fluctuations in glycemia and the amount of post-workout hypoglycemia. By analyzing the results of our examination, we exposed that the patients examined were equally prepared to perform each test in terms of carbohydrate, protein and fat intake. The test was performed 2 h before exercising. Patients were given a significantly different amount of carbohydrates, depending on the severity of the test effort and their energy demand. Despite this, no significant differences in blood glucose levels were observed in patients, both in the whole group and with respect to LFG and HFG. Based on the results of the study, it was concluded that the size of VO_2_ max in patients did not have a significant effect on carbohydrate intake and blood glucose levels after the completion of three exercise tests (Table 2). The lowest blood glucose levels were observed in patients after aerobic effort. In addition, patients with higher levels of VO_2_ max presented lower and more stable glucose levels 2 h before exercise as well as lower glycemic variability during the aerobic effort. We could also conclude that the effort characterized by mixed metabolism, especially with a predominance of anaerobic, often led to hyperglycemia in patients, which was observed during exercise and shortly after its completion (Figure 1).

The results of studies of other research centers are contradictory in relation to the degree of physical capacity of healthy individuals and type 1 diabetes. Komatsu et al. [31] suggested that young type 1 diabetic patients, as compared to healthy people, show lower oxygen capacity measured on the basis of maximal oxygen uptake (VO_2_ max). In contrast, Adolfsson et al. [10] stated that such groups do not differ to a large extent. On the other hand, Cuencia-Garcia et al. [19], who compared the results of 8–16-year-old children with diabetes with those of their siblings, did not show any difference between the studied groups in terms of their efficiency and physical activity. In the literature review process, we did not find studies involving the use of the latest glycemic monitoring systems (CGMs) during various types of exercise in young patients, but only in adults [32,33]. In our view, an element of optimal therapy for diabetes in children is glycemic self-monitoring, based on a continuous blood glucose monitoring system (continuous glucose monitoring, CGM, with up to 288 measurements per day). With its application, the patient can observe the current glycemic level in real time and a graph which illustrates its changes over time. In addition to the numerical value, there are trend arrows indicating the direction and rate of glycemic changes [34]. CGM RT (continuous glucose monitoring real-time) systems, on the basis of sensor readings, actively inform about undesirable events and health threats to the patient (including hypo- and hyperglycemic alarms, rapid rate of glucose changes and a predictive early warning alarm of hypoglycemia), which allows the patient to take immediate corrective actions [35]. With this glycemic control method, it is possible to observe the variability of glycemia, identify unconscious hypoglycemia when the patient does not feel a decrease in blood glucose level below 70 mg/dL and observe fluctuations in glycemia, e.g., during exercise [36,37].

In our experimentation, with the application of CGM in the examined patients, in those with functioning devices, we were able to identify hypoglycemia that could have been omitted using, for example, a glucometer. Glycemic results obtained from the glucometer give point information and are therefore insufficient to detect short-term hyper- and hypoglycemic episodes or asymptomatic episodes.

The results of previous clinical trials on the application of continuous glycemic monitoring systems in type 1 diabetic patients are unequivocal in relation to the group of patients taking glucose measurements with glucose meters. They show significant limitations of daily blood glucose fluctuations and increased time spent by patients with normoglycemia [38]. Whereby the important role of normoglycemia in the first years of the disease in the prevention of late vascular complications, including kidney, visual and cardiovascular damage, is emphasized here. They are associated with the so-called phenomenon of “metabolic memory”, in which, after even a short period of hyperglycemia, e.g., during prolonged anaerobic effort, irreversible changes in gene expression occur, which determine the occurrence of distant vascular complications, even if in subsequent years of the disease glycemic normalization occurs [39]. It has also been shown that the fewer fluctuations there are in glycemia, the lower is the risk of acute complications, especially life-threatening hypoglycemia [40]. The value of our study was the inclusion of patients using continuous glucose monitoring systems treated with a personal insulin pump, which allowed continuous reading of glycemic levels and trends in glycemic changes during the clinical experiment. The identification of glycemic fluctuation, including hypo- and hyperglycemia in our patients, taking into account their level of physical performance and in relation to the type of exercise, has measurable clinical implications supporting the process of the patient’s treatment. The occurrence of hypoglycemia and the fear of hypoglycemia are still the greatest barriers to physical activity in people with type 1 diabetes. It is particularly difficult to maintain normal and stable glycemia during moderate to maximum activity. This type of physical activity can result in late hypoglycemia, including night hypoglycemia; currently, the most effective way to prevent it is through continuous glycemic monitoring systems integrated with insulin pumps that enable automatic suspension of insulin supply. Based on the results of the study, we found that patients with a higher VO_2_ max had a lower tendency for glycemic changes during the aerobic effort (Figure 2). Thus, it can be assumed that type 1 diabetic patients practicing endurance-oxygenic sports may have a better metabolic balance of diabetes and thus less risk of developing both acute and chronic complications of the disease.

Scott et al. [2] stated that oxygen metabolism effort, particularly long-term effort, can lead to hypoglycemia, not only during but also immediately after physical exercise as well as several hours afterwards. This can be explained by the metabolic mechanism in which a patient with type 1 diabetes, who does not have his own insulin, is also deprived of endogenous regulation responsible for maintaining a constant level of glycemia. Therefore, his muscles consume a large amount of muscle glycogen while doing their job, which results in the absorption of glucose directly from the bloodstream by muscle cells. Then, in the absence of external carbohydrate supply and at the same time a relatively high concentration of exogenous insulin, which prevents the “release” of liver glycogen and gluconeogenesis from fatty acids, a dramatic drop in blood glucose levels may occur. In contrast, in people without diabetes, insulin secretion decreases during exercise and the secretion of counter-regulatory hormones, which increase the production of glucose by the liver, balances the glucose uptake by skeletal muscles during exercise. This precise autonomic and hormonal regulation ensures that blood glucose levels remain stable during most types of physical activity [41]. In our studies, we showed that mixed effort can lead to short-term hyperglycemia immediately afterwards. This phenomenon is explained by increased concentrations of hormones secreted under the influence of intense short-term effort: adrenaline, cortisol, glucagon and growth hormone [25]. These hormones act in the opposite way to insulin and increase the processes of gluconeogenesis and glycogenolysis.

Identifiable limitations of the current study concern the low number of adolescents who participated in the research. Additionally, not all continuous glucose monitors provided good-quality data, which additionally reduced the sample in CGM analysis. Future research should investigate not only single physical exercises, but also reactions and adaptations for long-term training processes in type 1 diabetes.

## 5. Conclusions

Our research suggests that any patient with type 1 diabetes can perform physical activity but should remain under medical supervision and use a continuous glycemic monitoring system. By implementing this method, we exposed that different forms of physical activity have different effects on the level of glycemia; however, with continuous monitoring of glycemia, it does not prevent aerobic (e.g., endurance running training), mixed (team games) and anaerobic (weightlifting and explosive exercises) activity. Their aim is to increase by about 20% the level of aerobic capacity determined by the VO_2_ max index. After that, higher intensity exercises which increase the effect of regulating exercise and post-exercise glycemia can be started, which increase the effect of regulating exercise and post-workout hyperglycemia. Our results clearly indicate the direction of further studies on the influence of exercise of different intensity on glycemic changes in patients with type 1 diabetes. It is necessary to study the influence of 8–12 weeks of training interventions among numerous groups of patients representing different age groups and the state of the disease.

## Figures and Tables

**Table 1:** Characteristics of the study group (All) and subgroups with lower (LFG) and higher (HFG) VO_2_ max.

Characteristics	All (*n* = 20)	LFG (*n* = 10)	HFG (*n* = 10)	*p*-Value
Age (years)	14.3 ± 1.6	14.0 ± 1.7	14.7 ± 1.5	0.4076
Diabetes duration (years)	6.7 ± 4.1	6.1 ± 3.3	7.2 ± 4.9	0.5660
CSII (Continuous Subcutaneous Insulin Infusion) duration (years)	6.0 ± 3.9	5.3 ± 3.2	6.7 ± 4.5	0.4330
HbA1c (%)	7.3 ± 0.8	7.4 ± 0.8	7.2 ± 0.9	0.6078
DDI (UI/kg)	0.9 ± 0.3	0.9 ± 0.3	0.9 ± 0.3	0.5894
BMI z-score	0.2 ± 0.7	0.5 ± 0.8	−0.2 ± 0.5	0.0401
Body fat (%)	14.7 ± 6.4	19.0 ± 6.2	10.5 ± 2.8	0.0010
WHR (Inches)	0.8 ± 0.0	0.81 ± 0.04	0.78 ± 0.03	0.0254
VO_2_ max (mL/kg/min)	40.2 ± 5.8	36.2 ± 5.3	44.3 ± 2.5	N/A
TSH (uU/mL)	1.4 ± 0.8	1.3 ± 0.5	1.6 ± 1.0	0.7912 ^#^
FT4 (pmol/L)	11.9 ± 1.1	12.0 ± 1.1	11.9 ± 1.1	0.8342
Alt (U/L)	19.3 ± 7.6	18.7 ± 4.3	19.8 ± 10.0	0.7905 ^#^
Ast (U/L)	15.0 ± 4.2	15.2 ± 4.7	14.7 ± 3.8	0.7975
TC (mg/dL)	160.0 ± 25.5	171.7 ± 18.9	148.3 ± 26.6	0.0361
HDL (mg/dL)	60.4 ± 16.4	68.8 ± 14.1	52.0 ± 14.4	0.0170
LDL (mg/dL)	86.9 ± 21.0	91.3 ± 22.7	82.4 ± 19.1	0.3562
TG (mg/dL)	66.5 ± 35.7	61.8 ± 20.9	71.2 ± 47.0	0.9397 ^#^

^#^ Mann–Whitney U test was used due to lack of normal distribution.

**Table 2:** Comparison of glucose levels and consumed carbohydrates during each tests.

Type of Test	Maximum			Mixed			Aerobic			ANOVA *p*-Value	
Subgroups Regarding to VO_2_ Max	LFG (*n* = 10)	HFG (*n* = 10)	All (*n* = 20)	LFG (*n* = 10)	HFG (*n* = 10)	All (*n* = 20)	LFG (*n* = 10)	HFG (*n* = 10)	All (*n* = 20)	Type of Test	VO_2_ Max
Carbohydrates consumed before tests (g/kg)	0.20 ± 0.18	0.34 ± 0.33	0.27 ± 0.27	0.16 ± 0.18	0.24 ± 0.19	0.20 ± 0.18	0.36 ± 0.26	0.28 ± 0.31	0.32 ± 0.28	0.3677	0.4410
Starting glycemia (mg/dL)	159.70 ± 37.84	153.60 ± 40.74	156.65 ± 38.39	165.30 ± 24.78	149.30 ± 35.00	157.30 ± 30.63	140.00 ± 28.78	143.80 ± 22.13	141.90 ± 25.06	0.2663	0.4447
Carbohydrates consumed during and after tests (g/kg)	0.18 ± 0.34	0.05 ± 0.12	0.12 ± 0.26 ^#^	0.22 ± 0.26	0.13 ± 0.19	0.18 ± 0.23 ^$^	0.46 ± 0.23	0.33 ± 0.29	0.40 ± 0.27 ^# $^	0.0003	0.1896
Last glycemia during tests (mg/dL)	165.80 ± 32.79	145.70 ± 28.83	155.75 ± 31.77 ^#^	136.40 ± 39.81	129.80 ± 34.50	133.10 ± 36.41	118.70 ± 50.20	110.70 ± 34.57	114.70 ± 42.15 ^#^	0.0011	0.3483
Glycemia at rest (mg/dL)	158.22 ± 34.66	143.00 ± 40.70	150.21 ± 37.73 ^# $^	120.90 ± 31.73	122.50 ± 33.13	121.70 ± 31.58 ^#^	102.00 ± 51.96	107.50 ± 35.92	104.75 ^$^ ± 43.56	0.0021	0.9433

^#^ and ^$^ significant pairs.

## Data Availability

The data presented in this study are available on request from the corresponding author.
